# Characterization of the External Load of Soccer Goalkeepers Depending on the Category and Sports Context

**DOI:** 10.3390/sports12120318

**Published:** 2024-11-26

**Authors:** Víctor Hernández-Beltrán, Boryi A. Becerra-Patiño, Abian Perdomo-Alonso, Jesús Barguerias-Martínez, Sergio Gómez-Carrero, Mário C. Espada, José M. Gamonales

**Affiliations:** 1 Training Optimization and Sports Performance Research Group (GOERD), Faculty of Sport Science, University of Extremadura, 10001 Cáceres, Spain; martingamonales@unex.es; 2 Faculty of Physical Education, National Pedagogical University, Bogotá 110221, Colombia; babecerrap@pedagogica.edu.co; 3 Management and Pedagogy of Physical Activity and Sport (GPAFD), Faculty of Physical Education, National Pedagogical University, Bogotá 110221, Colombia; 4 Real Madrid C.F., Valdebebas, 28055 Madrid, Spain; abianperdomo@gmail.com (A.P.-A.); jesus_bargueiras@hotmail.com (J.B.-M.); sergiete_5_1996@hotmail.com (S.G.-C.); 5 Comprehensive Health Research Centre, University of Évora, 7002-554 Évora, Portugal; mario.espada@ese.ips.pt; 6 Escola Superior de Educação, Instituto Politécnico de Setúbal (IPS), 2910-761 Setúbal, Portugal; 7 Sport Physical Activity and Health Research and Innovation Center (SPRINT), 2040-071 Rio Maior, Portugal; 8 Life Quality Research Centre (CIEQV), 2910-761 Setúbal, Portugal; 9 CIPER, Faculdade de Motricidade Humana, Universidade de Lisboa, 1600-214 Lisboa, Portugal; 10 Faculty of Education and Psichology, University of Extremadura, 06071 Badajoz, Spain

**Keywords:** sport, team, football, performance, goal

## Abstract

Background/Objectives: Studies focused on the soccer goalkeeper position in training and official matches are scarce. The present study aimed to analyze the external load during training sessions and official matches in semi-professional goalkeepers. Methods: Data from goalkeepers (n = 6) from the youth ranks of a professional team belonging to the first Spanish soccer league have been used. The sample is made up of a total of 758 data collected during all the training and competitions carried out by the analyzed teams that made up the squad during the 2021/2022 and 2022/2023 seasons. A descriptive and inferential analysis was carried out based on the category (Youth B or Youth C) and the sports context (training or competition). Results: The results showed significant differences depending on the category (average time to feet left, average time to feet right, total jumps, total dives, total left dives, total right dives, high metabolic load distance (HMLD), and high metabolic power efforts (HMPE)), and the sport context (average time to feet right, total jumps, total dives, total left dives, total right dives, total distance, distance 18–21 km/h, distance 21–24 km/h, Dec 2–3, efforts, and HMLD). Conclusions: The EL of the GKs shows differences regarding the category and the context. Therefore, it is necessary to analyze and determine the threshold of each player considering different variables related to the external and internal load to individualize the training tasks and prevent injuries due to overload.

## 1. Introduction

Football is a complex sport that demands high levels of physical, physiological, technical, and tactical responses [1]. Physical demand assessment has been used to determine that goalkeepers (GKs) of teams at low positions cover a greater distance in sprint actions (+372 m), defining how physical aspects are determinants in collective performance [2]. On the other hand, physical demand comprises the quantification of physical performance from external load (EL) variables such as total distance covered (TD), distance covered at various intensities, accelerations (ACC), decelerations (DEC), PlayerLoad™ [3], and internal load (IL), heart rate (HR), creatine kinase (CK), and Hooper index [4,5,6]. Some studies have also evaluated tactical responses based on the use of global positioning systems (GPS). GKs need specific reference zones for their performance, which are related to the physical actions they develop [7,8,9]. Therefore, it is important to quantify the specific demands during the competition to design training tasks that respond to the requirements of the matches [10].

The different technological advances have allowed for the implementation of increasingly relevant assessments, adjusted to the competitive reality, and highlighting the incorporation of technologies such as the local position system (LPS) based on ultra-wide band (UWB) [11], inertial measurement units (IMU) [12], and electronic performance systems (EPTS) [13]. These tools have allowed for performance assessments in association with the demands of the playing position [14]. In particular, one of the important goals for the technical staff professionals of teams is to monitor the physical and physiological demands of GKs, because this specific position has been evolving. Therefore, evaluating these players’ responses in training and competition can help to foster individualized sport preparation approaches focused on the true needs of the GKs and their competitive environment [15,16]. Currently, knowledge of the physical demands of GKs is still limited [17,18], highlighting the need to develop studies that consider the assessment of IL and EL in professional GKs.

The average TD of football GKs is between 5.6 and 6 km, of which 4025 ± 440 m is walking, 1223 ± 256 m is jogging, 291 ± 90 m is running, and 11 ± 12 m is sprinting [19]. The specific physical demands of GKs indicate that, in particular, the aerobic capacity over long distances does not influence their performance, while sprinting, jumping, and agility are determining factors [20]. These abilities are associated with other explosive actions that require jumping, diving, ACC, and DEC [21]. In that same pathway, some studies have reported that GKs only cover 50% of the TD when compared to the other football field positions [22], experiencing a series of short sprints of moderate intensity [23]. Consequently, and due to the nature of their position and the playing area GKs occupy, these players do not perform a high number of actions at maximum speed [24].

However, recent studies, such as the one developed by Vladovic et al. [25] using a Vector G7 device (Catapult Sports Ltd., Melbourne, Australia), assessed TD and found that GKs performed at high intensity (>5.5 m/s), as well as ACC (>3 m/s^2^) and maximum DEC (−3 m/s^2^). These measures respond to the specific needs of the football position [26], which is why they are increasingly incorporated into GK assessment. On the other hand, the study developed by White et al. [21] revealed that the height of the jump determines the specific performance of this position; these include high jumps with heights > 0.4 m, medium jumps between 0.2 and 0.4 m, and low jumps < 0.2 m. Commonly, the assessments performed for physical demand in football are linked to the number of pre- or post-match days (MD): among them, MD-4, MD-3, MD-2, and MD-1 are the pre-game sessions, and MD + 1 is the post-match session [23]. The study by Moreno-Pérez et al. [27] quantified the EL in professional GKs and found that there is a tendency to progressively reduce the amount of ACC and high-intensity DEC from MD-4 to MD-1; and in general, MD-2 was associated with being the day with the lowest EL. Likewise, it has been reported that monitoring seasonal training load (TL) reduces TD and PlayerLoad™ on MD-1 and MD + 1 [23]. Meanwhile, a recent study developed by Casamicha et al. [28] compared the EL of professional GKs in various sessions of a microcycle, concluding that there is a decrease in EL variables concerning the proximity of the next match. In contrast, GKs manifested a higher external training load (ETL) in MD-3 and a minimum in MD-2 and MD-1 [25]. Therefore, an adequate distribution of the training load is important to be successful in football [29,30,31].

Despite the existence of several research studies assessing the physical demand of GKs, not enough progress has been made in deeply understanding the differences and similarities with respect to the demand between training and official matches. Some studies have analyzed IL and EL in different microcycles with training and official matches [32], as well as the influence of pitch size and GKs on other players’ EL and IL measures [33]. However, although there is an increasing number of studies focused on the physical and physiological demands of goalkeeping, to date, no studies have been found that identify the most representative IL and EL indicators in professional GKs when comparing their performance in training and competition. Therefore, the present study aimed to analyze and compare the EL of professional GKs according to different contexts (training or match) and categories (Youth B and Youth C).

## 2. Method and Materials

### 2.1. Design

In the present study, a quasi-experimental, retrospective, and longitudinal design of the data [34] was carried out; an analysis of the data collected during the 2021–2022 and 2022–2023 seasons was carried out to characterize the performance of GKs under different situations (context and category). This analysis has been developed in previous studies which analyzed different variables in football [3].

### 2.2. Sample

The study sample consisted of a total of 758 cases collected over two full football seasons. Furthermore, the sample was divided between analysis of actions in training (*n* = 656) and in matches (*n* = 102). Data were collected from six GKs from the Real Madrid youth academy (age: 19.33 ± 0.81 years; height: 1.85 ± 0.04 cm; weight: 72.33 ± 3.61 kg; and experience: 3.34 ± 1.18 years), belonging to the Youth B (*n* = 618) and Youth C (*n* = 140) football squads.

### 2.3. Variable Codification

For the analysis of the data, a series of variables have been selected as determinants of GK performance according to the different analyzed categories or contexts. These variables were chosen due to are the most representative and important variables which can reveal differences in sports performance. Therefore, the following variables were selected for the study:

- Independent variables:
  ∘ Category (Youth B and Youth C);
  ∘ Context (Training and Match).
- Dependent variables:
  ∘ Flight time with the left foot;
  ∘ Flight time with the right foot;
  ∘ Total jumps;
  ∘ Total saves;
  ∘ Total saves on the left side;
  ∘ Total saves on the right side;
  ∘ Total distance;
  ∘ Distance between 18–21 km/h;
  ∘ Distance between 21–24 km/h;
  ∘ Distance > 24 km/h;
  ∘ Accelerations 2–3 m/s^2^;
  ∘ Accelerations > 3 m/s^2^;
  ∘ Decelerations 2–3 m/s^2^;
  ∘ Decelerations > 3 m/s^2^;
  ∘ Efforts;
  ∘ High metabolic load distance: This variable refers to the distance covered with an energy consumption greater than 25.5 W/kg [35]. Specifically, this value corresponds to running at a constant speed of 5.5 m/s or 19.8 km/h. ACC or DEC, for example, accelerate from 2 to 4 m/s^2^. This variable provides global information regarding the total high-intensity activities of players as it includes not only high-speed running but also ACCs and DECs, which justifies its analysis in football [3];
  ∘ High metabolic power efforts (HMPE): The cost of effort expended during ACC and DEC actions [36].

### 2.4. Instruments

To examine the anthropometric characteristics (age, height, and weight) of the GKs, a wall-mounted measuring rod (SECA, Hamburg, Germany) and a portable body composition monitor consisting of 8 contact electrodes (model BC-601, TANITA, Tokyo, Japan) were used. A total of 6 inertial devices (Catapult OptimEye S5, Catapult Innovations, Team Sport 5.0, Melbourne, Australia) were used to collect IL and EL data from the players [37]. Each player was fitted with a device to quantify all their movements, which was located in the interscapular area through an anatomical harness.

Data were processed with specific software (Catapult OpenField Version 3.1.0) and exported to Microsoft Excel sheets (2013 version: Microsoft Corporation, Redmond, WA, USA) for further analysis.

### 2.5. Procedure

To carry out the study, the club’s board of directors were contacted, and we also conducted a meeting with the coaching staff of the teams. In this meeting, the benefits and disadvantages associated with the research were explained. After obtaining approval for the data collection, the players’ legal representatives were provided with an informed consent form to sign. This was to ensure they understood and accepted the various phases of the research.

Subsequently, a phase of familiarization of the players with the devices that were going to be used in the collection was carried out, with the aim of not influencing the performance and physical capacity of the GKs. This familiarization was conducted during a training session in which all the players wore the anatomical harness to prevent chafing and discomfort with clothing. Finally, the data were collected during the different training sessions and matches in which the club participated during the two seasons. The training sessions were focused on improving the technical and tactical skills of the players according to different situations (offensive and defensive actions) and constraints (vision limitations with barriers, penalties, free kicks, or superiority in attacks). These training sessions were divided into three main parts: warm-up, main part, and cool-down. The main part of the session focused on improving individual and group skills. The training sessions had an average of 1.30 h.

The present study was developed under the premises of the Declaration of Helsinki [38], as well as considering the ethical standards for the development of research in the field of sports science [39]. Moreover, this study was approved by the Bioethics Committee of the University of Extremadura (67/2017).

### 2.6. Statistical Analysis

First, an assumption analysis was carried out. The Kolmogorov–Smirnov test [40] was used to identify the normality of the data, obtaining a normal distribution. Parametric models were used (students’ *t*-tests) for the data analysis. Then, a descriptive analysis of the sample was carried out to characterize the data by means of frequencies (mean and standard deviation). Afterwards, the student’s *t*-test was performed to compare the data obtained according to category (Youth B or Youth C) and context (training or competition). Finally, effect size (ES) was calculated considering Cohen’s d proposal: insignificant: <0.20; small: 0.20–0.50; medium: 0.51–0.80; and large: >0.80 [41].

IBM SPSS Statistics for MAC OS (version 27, 2021, IBM Co., Ltd., Armonk, NY, USA) was used for statistical analyses. Statistical significance was determined at *p* < 0.05.

## 3. Results

Descriptive results, as well as the results of the variance test for each sample, are shown in Table 1; Table 2 to analyze whether there are differences depending on the category of the GKs (Table 1) and the context analyzed (Table 2). Considering the category analysis, it is shown that four variables presented a high ES (average time to feet left, average time to feet right, HMLD, HMPE), with a significance value of *p* < 0.001.

Taking into account the context, only one variable presented a high ES (total distance), with higher values during the matches than in the training sessions.

## 4. Discussion

This study aimed to conduct a systematic analysis under different contexts (training and competition) and categories (Youth B and Youth C) of the EL to which the GKs of a development football team were exposed. For this purpose, different variables of EL have been analyzed to quantify and monitor the physical demands during training and competition periods. The results supported that there are significant differences depending on the analyzed context, with the magnitude of the effect ranging from small to insignificant. The load on GKs has been analyzed during different microcycles, consisting of training periods and official matches, obtaining higher values of ACC and DEC in those short training periods, with the duration of the microcycles not affecting the load during official matches [32]. Therefore, the present study is valuable for football sports technicians, especially goalkeeper specialists, since two complete football seasons with their corresponding training sessions and matches are analyzed.

The use of the GK in training represents an influential variable during the development of reduced games or small-sided games (SSG) because his presence, or not, allows for the modification of the physical demands of the players during the tasks [33]. Therefore, the figure of the GK should be considered one of the most important both during training and in competitions as it allows the physical demands of the outfield players to be modified to a large extent. In addition, the gender of the GK should be taken into consideration as it has a significant influence on the loads during training, with male GKs being slightly exposed to superior loads considering variables such as TD, maximum speed, and the number of ACC and DEC [42]. Also, the soccer goalkeeper has different characteristics compared to any other field player because of the role he plays during the game, which contains a high degree of responsibility [43]. In recent years, the position has experienced an evolution at the technical–tactical level conditioned by regulatory modifications, adding to the interest of coaches in the goalkeeper acting as the last line of defence and the first attacker in the construction of the offensive game [44,45]. Studies such as this work are essential since they allow us to determine which EL variables influence the development of soccer GK functions. Following the results obtained, it is easy to identify which variables influence the sports performance of the GKs according to their category or context analyzed. Therefore, the coaching staff can analyze which parameters must be developed to improve their performance and physical fitness.

Taking as a reference the category to which the analyzed GKs belong, there are differences in 8 of the 15 analyzed variables. After observing the size of the effect produced by the differences, it is the variables “Average Time to Feet Left”, “Average Time to Feet Right”, “HMLD”, and “HMPE” that present the largest ES. The Youth B GKs obtained significantly higher values in the first two variables, and Youth C GKs developed higher results in the rest of the variables. The “HMLD” and “HMPE” are variables derived from the different movements and ACC and DEC produced at high intensity [37], representing a significant increase in fatigue and risk of injury in these players [46]. These variables, as well as the physiological adaptability of GKs to high-intensity actions, are the most significant determining factors for the development of training strategies [47]. The results related to the EL of GKs can be very useful for coaches and can, at the same time, increase the quality of training programs in the learning stages of football.

Therefore, certain physical qualities, such as strength, agility, and speed, should be worked on daily in a precise and controlled manner during both training and during competition [48] due to the fact that if these players show better values in those variables, the sports performance will be increased under different situations, i.e., long matches with high values of EL (extra time). In the same way, the GK figure is a game variable that allows for the modification of the physical demands of the outfield players in different training formats of SSG, with his presence leading to higher ACC values and high-intensity actions performed by the outfield players when compared to ball possession SSGs [48,49]. Knowing the intensity during training would allow coaches to evaluate each technical–tactical skill under the same intensity conditions as in the competition.

Concerning the context, the kinematic variables present higher values during competition, a fact that we relate with the high-intensity actions. On the contrary, the variables that concern the time on the left or right foot, as well as the number of saves, are higher in the training environment due to the high volume of actions that are performed during this period to improve the technical–tactical aspects of the game. In this sport, players must possess cognitive, perceptual, and motor skills that can adapt to high-intensity situations and in-game changes, regardless of their position [50]; their importance increases when considering the figure of the GKs [44]. Football training has an important role in the acquisition and improvement of technical skills, as well as techno-motor aspects. Therefore, it is essential to teach technical–tactical gestures from an early age to future soccer GKs and to adapt training to the demands of the competition.

The GKs develop a series of characteristics and functions that are different from the rest of the players in football due to the role and position they assume in the game [43]. This fact forces them to develop technical actions such as deflections, clearances, rejections, or saves [51,52], as well as physical skills, such as reaction speed, agility, explosive strength, and flexibility [53]. Considering this evidence, the coaching staff should consider the physical demands that occur in competition to be able to match this load in training sessions and specific tasks planned for the GKs [54]. The findings of the study can serve football professionals in the process of prescribing specific training for GKs. Based on the current results, they show that there are significant differences depending on the category (Youth B or Youth C) and the sports context (training or competition).

Therefore, analyzing the EL over two seasons can be useful since it allows us to determine the optimal performance of the GKs. On the other hand, the main limitation of the study was the small sample we have had access to; only six GKs have been analyzed throughout the two seasons in both training and competition situations. This fact limits the possibility of extrapolating the data to the rest of the GKs and teams. Therefore, for future studies, it is recommended to obtain more extensive data and access to more seasons in order to be able to generalize the results and draw objective and empirical conclusions.

## 5. Conclusions

The present study showed the existence of differences in the EL of GKs during two seasons depending on the category (average time to left feet, average time to right feet, total jumps, total dives, total dives to the left, total dips to the right, HMLD, and HMPE) and the context (average time to feet right, total jumps, total jumps left, total jumps right, total distance, distance 18–21 km/h, distance 21–24 km/h, Dec 2–3, efforts, and HMLD). In summary, the EL of the GKs shows differences regarding the category and the context. Therefore, it is necessary to analyze and determine the threshold of each player considering different variables related to the external and internal load to individualize the training tasks and prevent injuries due to overload. Understanding the intensity during training helps coaches assess each technical–tactical skill under the same conditions as in competition.

Also, the present study is one of the first to focus on analyzing the physical demands of GKs in a football youth academy in order to understand in greater depth the functionality and adaptability of these specific players to training and competition conditions, as well as identify whether there are differences between the category and the context. Studies like this are essential for identifying which EL variables influence the development of soccer goalkeepers’ functions.

## 6. Practical Application

Analyzing the load to which the athletes are exposed allows for the identification of the peaks of maximum performance, as well as the situations of maximum physical demand, and for adaptation to the TLs to improve the performance of the athletes in situations of high physical and cognitive stress, while preventing, in parallel, injuries occurrence. Therefore, in future studies, it is recommended to quantify the motor performance of GKs under different game situations or constraints, such as penalty kicks, and attacking actions in situations of numerical inferiority or superiority, defensive actions, or corner kicks. Another aspect to be studied is the constructive phase of the game, in which GKs are involved in modern soccer through their movements and footwork.

## Figures and Tables

**Table 1:** Descriptive and inferential analysis considering the category.

Variables	Youth B				Youth C				F	Sig.	t	df	*p*	Cohen’s d	ES
	Mean	SD	(95% CI)		Mean	SD	(95% CI)								
			Lower	Upper			Lower	Upper							
Average Time to Feet Left	1.33	1.00	1.26	1.42	0.13	0.46	0.06	0.22	51.71	0.000	13.83	756	0.000 *	1.29	High
Average Time to Feet Right	1.33	0.88	1.26	1.40	0.13	0.50	0.05	0.22	83.41	0.000	15.67	756	0.000 *	1.47	High
Total Jumps (n)	17.28	16.93	15.91	18.64	23.67	19.13	20.54	26.99	4.11	0.043	−3.94	756	0.000 *	−0.37	Small
Total Dives (n)	16.61	41.94	14.09	20.69	2.41	11.29	0.87	4.74	9.44	0.002	3.97	756	0.000 *	0.37	Small
Total Left Dives (n)	7.72	23.54	6.33	10.10	1.26	6.29	0.44	2.52	6.70	0.010	3.22	756	0.001 *	0.30	Small
Total Right Dives (n)	8.03	18.66	6.88	9.78	1.14	5.33	0.40	2.15	13.32	0.000	4.32	756	0.000 *	0.40	Small
Total Distance (m)	2668.73	1263.24	2572.10	2775.71	2830.90	1212.79	2635.47	3032.66	0.07	0.791	−1.38	756	0.168	−0.13	Insignificant
Distance 18–21 km/h (m)	6.83	11.07	5.98	7.74	9.07	19.09	6.40	12.66	2.56	0.110	−1.85	756	0.065	−0.17	Insignificant
Distance 21–24 km/h (m)	3.09	11.17	2.31	4.04	2.93	11.24	1.37	5.03	0.12	0.734	0.15	756	0.880	0.01	Insignificant
Distance > 24 km/h (m)	3.11	20.60	1.63	4.93	0.49	2.28	0.16	0.92	8.22	0.004	1.50	756	0.133	0.14	Insignificant
ACC 2–3 (m/s^2^)	15.38	9.19	14.68	16.10	16.09	9.80	14.48	17.87	1.27	0.261	−0.82	756	0.415	−0.08	Insignificant
ACC > 3 (m/s^2^)	4.15	5.22	3.77	4.60	3.91	3.61	3.33	4.60	1.89	0.170	0.51	756	0.611	0.05	Insignificant
DEC 2–3 (m/s^2^)	11.55	7.55	10.95	12.13	12.51	8.82	11.10	14.08	6.22	0.013	−1.32	756	0.186	−0.12	Insignificant
DEC < −3 (m/s^2^)	3.89	4.45	3.53	4.28	4.68	4.89	3.88	5.53	3.59	0.059	−1.85	756	0.065	−0.17	Insignificant
Efforts	66.95	93.02	61.31	75.42	63.46	37.62	57.22	70.16	0.10	0.746	0.44	756	0.662	0.04	Insignificant
HMLD	111.58	60.87	106.90	116.74	251.39	203.61	217.16	282.24	329.97	0.000	−14.48	756	0.000 *	−1.36	High
HMPE	24.15	14.17	23.10	25.27	91.57	68.88	80.41	103.28	701.76	0.000	−22.38	756	0.000 *	−2.09	High

**Note.** * *p* < 0.05; ACC: accelerations; DEC: decelerations; HMLD: high metabolic load distance; HMPE: high metabolic power efforts; SD: standard deviation.; df: degree of freedom.

**Table 2:** Descriptive and inferential analysis according to the context.

Variables	Training				Match				F	Sig.	t	df	*p*	Cohen’s d	ES
	Mean	SD	(95% CI)		Mean	SD	(95% CI)								
			Inferior	Superior			Inferior	Superior							
Average Time to Feet Left	1.13	1.04	1.05	1.22	0.96	1.02	0.77	1.16	0.13	0.716	1.54	756	0.124	0.16	Insignificant
Average Time to Feet Right	1.14	0.93	1.06	1.21	0.93	1.01	0.74	1.12	0.01	0.936	2.04	756	0.042 *	0.22	Small
Total Jumps (n)	19.19	18.21	17.85	20.66	13.77	11.07	11.67	15.87	17.93	0.000	2.92	756	0.004 *	0.31	Small
Total Dives (n)	15.49	41.18	12.97	19.00	4.31	6.14	3.16	5.60	8.66	0.003	2.73	756	0.006 *	0.29	Small
Total Left Dives (n)	7.25	23.07	5.91	9.18	1.88	2.97	1.33	2.51	6.56	0.011	2.34	756	0.019 *	0.25	Small
Total Right Dives (n)	7.49	18.34	6.31	9.05	2.03	3.58	1.42	2.78	10.89	0.001	3.00	756	0.003 *	0.32	Small
Total Distance (m)	2490.27	1122.00	2406.65	2575.38	4039.07	1241.75	3783.69	4259.79	1.66	0.199	−12.78	756	0.000 *	−1.36	High
Distance 18–21 km/h (m)	6.10	12.72	5.18	7.16	14.61	11.94	12.28	17.04	4.54	0.033	−6.33	756	0.000 *	−0.67	Medium
Distance 21–24 km/h (m)	2.63	11.44	1.82	3.58	5.85	8.85	4.20	7.77	3.23	0.073	−2.72	756	0.007 *	−0.29	Small
Distance > 24 km/h (m)	2.87	20.01	1.52	4.59	1.07	2.80	0.55	1.65	3.58	0.059	0.91	756	0.365	0.10	Insignificant
ACC 2–3 (m/s^2^)	15.29	9.41	14.56	16.00	16.93	8.48	15.29	18.51	2.08	0.150	−1.66	756	0.096	−0.18	Insignificant
ACC > 3 (m/s^2^)	4.18	5.26	3.80	4.58	3.67	2.20	3.24	4.07	13.78	0.000	0.96	756	0.336	0.10	Insignificant
DEC 2–3 (m/s^2^)	11.47	7.93	10.85	12.08	13.38	6.77	12.16	14.72	6.16	0.013	−2.31	756	0.021 *	−0.25	Small
DEC < −3 (m/s^2^)	4.16	4.81	3.79	4.53	3.28	2.06	2.88	3.69	34.00	0.000	1.81	756	0.071	0.19	Insignificant
Efforts	71.13	90.75	65.86	79.21	35.29	17.17	31.94	38.72	5.63	0.018	3.98	756	0.000 *	0.42	Small
HMLD	133.77	117.90	123.99	143.28	160.79	104.85	142.07	182.46	1.18	0.277	−2.18	756	0.029 *	−0.23	Small
HMPE	36.26	41.95	33.10	39.85	38.81	38.39	32.22	46.46	1.77	0.184	−0.58	756	0.563	−0.06	Insignificant

**Note.** * *p* < 0.05; ACC: accelerations; DEC: decelerations; HMLD: high metabolic load distance; HMPE: high metabolic power efforts; SD: standard deviation; df: degree of freedom.

## Data Availability

The data supporting this study’s findings are available from the corresponding author, upon reasonable request.
